# Cutaneous Canine Mast Cell Tumor: The Use of Proliferative Markers (Ki-67 and Ki-67 × AgNOR) in Cytological Samples for Diagnosis and Prognosis

**DOI:** 10.3390/vetsci11010023

**Published:** 2024-01-07

**Authors:** Christina Marouda, Tilemahos Anagnostou, Barbara Brunetti, Ioannis Savvas, Lysimachos G. Papazoglou, Dimitra Psalla

**Affiliations:** 1 Laboratory of Pathology, School of Veterinary Medicine, Faculty of Health Sciences, Aristotle University of Thessaloniki, 54124 Thessaloniki, Greece; 2 Unit of Anaesthesiology and Intensive Care, Companion Animal Clinic, School of Veterinary Medicine, Faculty of Health Sciences, Aristotle University of Thessaloniki, 54627 Thessaloniki, Greece; 3 Department of Veterinary Medical Sciences, University of Bologna, 40064 Bologna, Italy; 4 Unit of Surgery and Obstetrics, Companion Animal Clinic, School of Veterinary Medicine, Faculty of Health Sciences, Aristotle University of Thessaloniki, 54627 Thessaloniki, Greece

**Keywords:** mast cell tumors, immunocytochemistry, Ki-67, Ki-67 × AgNOR, cytological grading, dog

## Abstract

**Simple Summary:**

This study reports on the use of Ki-67 and Ki-67 × AgNOR markers in cytological specimens of canine mast cell tumors. The research involved 45 dogs with cutaneous mast cell tumors. The cytological specimens were graded and assessed for these markers. The study suggests potential cut-off values for these markers in correlation with histopathological grading. The findings highlight the importance of cytological evaluation and the inclusion of Ki-67 and Ki-67 × AgNOR markers in assessing highly malignant mast cell tumors.

**Abstract:**

A cytological grading system for canine mast cell tumors (MCTs) has been developed, but its integration into clinical routine has been hindered due to its diagnostic limitations. The aim of this study was to assess the prognostic value of Ki-67 and argyrophilic nucleolar organizing region (AgNOR) markers in cytological MCT samples and to determine cut-off values for these markers in correlation with histopathological grading. Cytological samples were collected prior to surgical excision, and histopathological samples were obtained postsurgery from 45 dogs diagnosed with cutaneous mast cell tumors (MCTs). The cytological specimens were classified using a two-tier grading system, and their Ki-67 (average immunopositive nuclei per 100 cells) and AgNOR (average AgNOR counts per 100 nuclei) signaling was assessed. Through receiver operating characteristic (ROC) analysis, cut-off values for Ki-67 and Ki-67 × AgNOR were determined to better align with histopathological grading (classified as low or high grade according to Kiupel’s scoring system). Without the inclusion of proliferative markers, there was a 73% agreement between cytological and histopathological grading. The prediction of histopathological grade was slightly more accurate when assessing Ki-67 and Ki-67 × AgNOR signaling in cytological specimens (75% and 80%, respectively) compared to the initial cytological grading. The cytological assessment of canine MCTs proves beneficial for the initial evaluation, and the incorporation of the evaluation of Ki-67 and AgNOR markers may assist in identifying diagnostically highly malignant MCTs.

## 1. Introduction

Mast cell tumor (MCT) is a common cutaneous tumor occurring at a frequency of 7–21% in dogs. These tumors exhibit a diverse range of biological behaviors; some may grow slowly, presenting a rather benign behavior, while others can emerge suddenly and grow very quickly with potentially life-threatening complications [1,2,3]. Wide surgical excision is considered front-line treatment for cutaneous and subcutaneous MCTs. The variability in the behavior of these tumors makes it challenging for oncologists to decide upon treatment modalities [1,4,5]. Grading of canine mast cell tumors (MCTs), beyond a simple morphological diagnosis, is the most important key for prognostic assessment and determination of treatment options [2,3,4,6]. Numerous studies have correlated cytologic and histopathologic characteristics of MCTs with their biological behavior, prognosis, and response to treatment [2,3,7,8,9,10,11]. Early determination of the grade would be crucial for surgical planning and clinical staging or the use of adjunctive therapy prior to surgery [5,6].

Cytology is a quick and easy method for a tentative diagnosis of mast cell tumors [7,8,12]. Despite the great diagnostic sensitivity of cytology, its use for canine MCT grading is debatable [13,14]. The inclusion of cytological grading in clinical practice has not been widely adopted as its association with clinical outcomes has not been extensively documented. Therefore, determination of the proper therapeutic approach is currently based on surgical biopsies [15]. Correlation of cytologic features with histopathological grade has been investigated in a few studies [7,8,16]. A cytological grading scheme using a two-tier histologic grading scheme (Kiupel’s histological grading system) as a gold standard has been developed by Camus et al. [7], and its applicability has been examined in one study [8]. Up to today, cytological grading with the aid of complementary proliferation markers has not been evaluated.

Prognostic indicators, such as Ki-67 and argyrophilic nucleolar organizing region (AgNOR), have been proven to be useful for histopathological grading of MCTs [6,10]. Distinct cut-off values have been proposed for Ki-67 and Ki-67 × AgNOR scoring for the classification of tumors as having low or high malignancy [6]. However, neither of these markers have been used on cytological specimens. The objective of this study was to assess the prognostic significance of the proliferative markers Ki-67 and AgNOR in cytological MCT samples and to define threshold values for these markers in correlation with histopathological grading. We hypothesized that grading of MCTs through the combined assessment of cytology and immunocytochemistry will improve the diagnostic ability to detect high-grade neoplasms.

## 2. Materials and Methods

### 2.1. Animals

This clinical study was conducted as a prospective, blinded, randomized cohort study. A total of 45 dogs diagnosed with cutaneous MCTs were brought to the Companion Animal Clinic of the Veterinary School, Aristotle University of Thessaloniki, Thessaloniki, Greece, during the period April 2018 to September 2020 for surgical excision of the tumors. As a component of the diagnostic procedure, fine-needle aspiration (FNA) specimens were collected from the lesions from all animals (designated as cytological samples C1) before the surgical excision. All dogs were subjected to hematologic examinations, serum biochemistry examinations, and a thorough preanesthetic clinical examination prior to surgery. Following the surgical procedure, which involved a wide surgical excision with 2 cm margins of healthy tissue conducted under general anesthesia, the entire excised masses were sent for histopathological examination (referred to as histopathological samples H2). The study described here adhered strictly to national and European animal welfare guidelines and received approval from the Institutional Ethical Committee (Approval Number: 567/13-3-2018).

### 2.2. Sampling

Multiple cytologic specimens were collected via FNA before surgery from all animals (3 for routine cytology, 3 for the Ki-67 index, 3 for AgNOR), while the final assessment was conducted on one sample for each technique that had a satisfactory number of intact and stained cells (at least 100 intact cells in monolayered areas). The cytologic samples (designated as samples C1) were fixed in a methanol solution. The surgically removed tumors (designated as samples H2) were fixed in 10% neutral buffered formalin and subsequently submitted for histopathologic evaluation.

### 2.3. Cytologic Examination and Grading

Cytologic samples from each case were stained with May–Grünwald–Giemsa (Sigma-Aldrich, Merck, Darmstadt, Germany). MCTs were classified according to Kiupel’s two-tier grading system proposed by Camus et al. [7] for cytologic specimens. In this system, samples exhibiting poor granulation and/or meeting at least two malignancy criteria (such as the presence of mitotic figures, nuclear pleomorphism, binucleation or multinucleation, or marked anisokaryosis) were classified as high grade. Conversely, well-granulated samples without the mentioned malignancy features were categorized as low grade.

### 2.4. Histopathological Examination and Grading

Slides were prepared with 4 μm thick sections of formalin-fixed paraffin-embedded specimens. The standard staining procedure with hematoxylin and eosin was applied. Histopathologic grading was performed based on Kiupel’s (low and high grade) grading system [2].

### 2.5. Immunocytochemistry and Ki-67 Scoring

Immunocytochemical staining and assessment for the proliferative marker Ki-67 was conducted in the Laboratory of Pathology, School of Veterinary Medicine of the Aristotle University of Thessaloniki, Thessaloniki, Greece. Immunolabeling for Ki-67 was performed on cytological samples using a monoclonal mouse antihuman antigen (Clone MIB-1, Dako, Glostrup, Denmark) at a dilution 1:50 for one hour following epitope retrieval in EDTA incubation (EnvisionFLEX, Target retrieval solution, pH 9, Dako, Glostrup, Denmark) for 20–30 min at 500 watts. The Ultravision Quanto Detection System HRP DAB (DAB Quanto chromogen, Epredia, Montréal, Quebec, Canada) was used for detecting the primary antibody binding, and smears (a subset of 45 samples suitable for evaluation) were counterstained with hematoxylin. For Ki-67 scoring, manual cell counting was performed in all samples. Areas with the highest proportion of immunocytochemically positive neoplastic mast cells were selected, and the total numbers of positive staining nuclei were calculated. Positive nuclei were counted in 100 cells in each sample, and mean values were calculated (immunopositive nuclei per 100 cells, ×400 magnification).

### 2.6. AgNOR Cytochemical Staining and Ki-67 × AgNOR Scoring

AgNOR staining on cytological samples was performed following the silver staining method introduced by Ploton et al. [17]. The counting of AgNORs was conducted in 100 randomly selected neoplastic mast cells and observed under ×1000 magnification. Nonmonolayered areas and areas where the neoplastic cells overlapped were excluded. AgNOR mean values per cell from the cytological samples were determined, and the product of Ki-67 × AgNOR was also calculated.

### 2.7. Grading Comparisons and Statistical Analysis

Based on immunocytochemical results (Ki-67 and Ki-67 × AgNOR), samples were classified into low or high grade using cut-off values determined in the present study. Cut-off values for Ki-67 and Ki-67 × AgNOR on cytological specimens were determined using receiver operating characteristic (ROC) analysis to achieve the highest possible accuracy in the prediction of the degree of malignancy by evaluating the cytological samples and furthermore to confirm their diagnostic validity.

Using Kiupel’s histopathological grade of H2 samples as a gold standard, we assessed the success rate of the cytological and immunocytochemical grade or their combination in relation to the histopathological one. Disagreements between cytologic, Ki-67, Ki-67 × AgNOR, and histopathologic gradings were noted, and the number of cases with grade discrepancies and the extent to which each of the above methods (cytologic, Ki-67, and Ki-67 × AgNOR gradings) predicted histopathologic grade were assessed. Furthermore, we tested if the above methods considered in combination detected highly malignant MCTs more accurately. All statistical analyses were performed using the software package IBM SPSS Statistics, version 27.

## 3. Results

### 3.1. Animals

The study included a total of 45 dogs, comprising 29 males and 16 females. Among them, 5 were mixed breed dogs and 40 were of pure breeds, including 7 Boxers, 6 Labradors, 5 Golden Retrievers, 5 Pit Bulls, 4 French Bulldogs, 3 English Setters, 3 Maltese, 2 Brittany Spaniels, 1 American Staffordshire terrier, 1 English Bulldog, 1 Pincher, 1 Pug, and 1 Yorkshire Terrier. The age of the dogs ranged from 2 to 15 years, and their body weight varied from 4 to 39.7 kg.

### 3.2. Comparison of Cytological and Histopathological Grading

**Figure 1:**
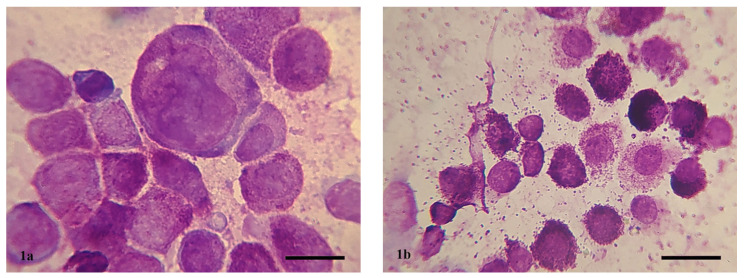
(**a**) High-grade MCT according to the cytological grading by Camus. The presence of large, round to ovoid cells was noted, with centrally or eccentrically located, round to ovoid basophilic nuclei exhibiting chromatin hypersegmentation in their cytoplasm. There was a significant degree of nuclear and cellular pleomorphism, anisocytosis, and anisokaryosis in >50% of nuclei, and neoplastic cells did not contain distinct cytoplasmic granules. (**b**) Low-grade MCT. The presence of large, round cells was noted, with centrally located, round nuclei exhibiting homogeneous chromatin distribution and numerous basophilic granules. Additionally, metachromatic granules were observed freely in the field. Bar = 15 µm.

Based on Camus’s cytological grading, 20 of the 45 MCTs (C1 samples) were classified as highly malignant (Figure 1a) and 25 as low malignant (Figure 1b). Based on Kiupel’s histopathological grading, 14 of the 45 MCTs (H2 samples) were classified as high grade and 31 as low grade. Cytology correctly diagnosed the histopathological grading in 33 out of 45 cases (22 low grade and 11 high grade). Specifically, 9 cases were misclassified as high grade and 3 as low grade by cytology. Cytological grading and histopathological grading are depicted in detail in Table S1 in Supplementary Materials.

### 3.3. Cut-Off Values for Ki-67 and Ki-67 × AgNOR

**Figure 2:**
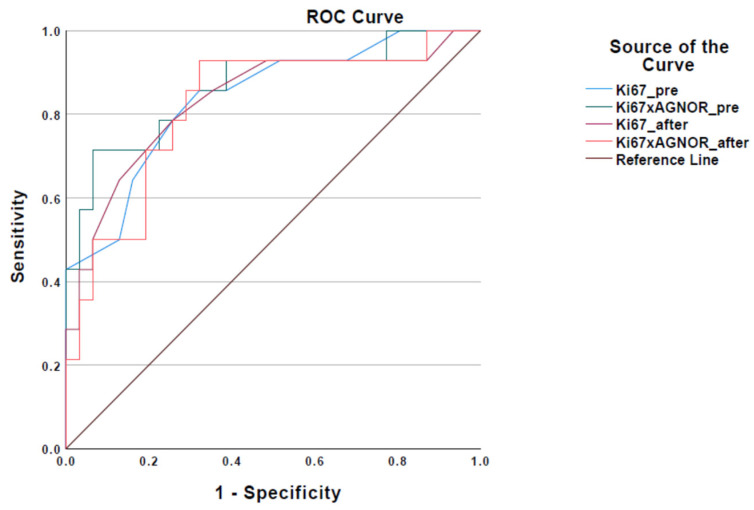
ROC curve for Ki-67 and Ki-67 × AgNOR values in C1 samples (Ki67_pre-curve: summary of mean Ki-67 values in C1 samples, Ki67 × AGNOR_pre-curve: summary of Ki-67 × AgNOR values in C1 samples).

The mean values obtained from the measurements of Ki-67 and AgNORs, as well as their product Ki-67 × AgNOR, in cytological samples are given in Table S2 in Supplementary Materials. Based on the numerical data listed in Table S2, the ROC curve method was applied for the comparative evaluation of Ki-67 and Ki-67 × AgNOR markers in C1 samples. The resulting ROC curves are depicted in Figure 2.

The area under each curve in Figure 2 corresponds to the extent of the accuracy with which each marker (Ki-67 and Ki-67 × AgNOR) obtained from the immunocytochemical and cytochemical examination can accurately diagnose the grade of malignancy (low or high) of an MCT as determined by histopathological examination and Kiupel classification (reference classification). Based on the results of the ROC curve method, markers Ki-67 (Ki67_pre-curve) and Ki-67 × AgNOR (Ki67 × AGNOR_pre-curve) obtained from the examination of the C1 samples could more accurately diagnose the degree of malignancy (low or high) of a MCT as determined by histopathological examination and Kiupel classification. Values 6.5 for the Ki-67 index and 15.46 for the Ki-67 × AgNOR index were selected as cut-off points, presenting the best possible combination of specificity and sensitivity (Supplementary Tables S3 and S4)

### 3.4. Comparison of Immunocytochemical (Ki-67 and Ki-67 × AgNOR) and Histopathological Grading

**Figure 3:**
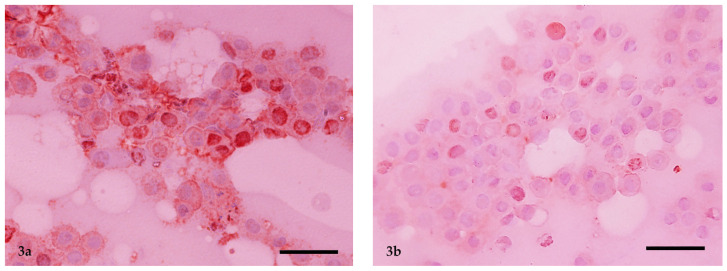
(**a**) MCT of high malignancy based on the Ki-67 score (>6.5) and (**b**) MCT of low malignancy based on the Ki-67 score (<6.5). Ki-67 immunopositive nuclei of neoplastic mast cells were observed. Immunocytochemical method. Bar = 25 µm.

Based on the Ki-67 cut-off value of 6.5, 19 of the 45 MCTs were classified as highly malignant (Figure 3a) and 26 as low malignant (Figure 3b). Immunocytology of Ki-67 correctly diagnosed the histopathological grading in 34 out of 45 cases. Specifically, 8 cases were misclassified as high grade and 3 as low grade by immunocytology.

**Figure 4:**
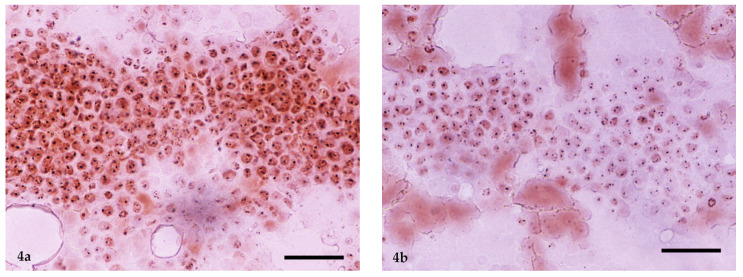
(**a**) MCT with high mean AgNORs/cell and (**b**) MCT with low mean AgNORs/cell. Argyrophilic organizing regions of the nucleus of neoplastic mast cells are depicted as brown-like granules of variable size. AgNOR histochemical staining. Bar = 25 µm.

Based on the Ki-67 × AgNOR cut-off value of 15.46, 18 of the 45 MCTs were classified as highly malignant (Figure 4a) and 27 as low malignant (Figure 4b). Applying the Ki-67 × AgNOR marker on cytological smears correctly diagnosed the histopathological grading in 35 out of 45 cases. Specifically, 7 cases were misclassified as high grade and 3 as low grade by Ki-67 × AgNOR scoring.

All numerical data obtained from the measurements of the Ki-67 and Ki-67 × AgNOR markers as well as the classification into low and high grade based on cut-off values are depicted in Table S5 in Supplementary Materials.

Table 1 summarizes all the assigned grades based on the histopathological, cytological, and immunocytochemical examination as well as the misclassified cases by each method.

## 4. Discussion

In routine clinical practice, the initial diagnosis of a MCT case is made by cytology of the macroscopically observed mass. The usual tactic is to aspirate the mass (FNA), a noninvasive procedure that has fewer side effects compared to surgical excision [6,14]. Despite the high diagnostic sensitivity of cytology, the lack of reliable grading makes it difficult to develop an appropriate therapeutic approach without surgical biopsy.

To assess the diagnostic value of cytologic grading in the current study, all cytologic specimens underwent assessment using the Camus grading system. This system is an adaptation of the Kiupel grading system, which classifies tumors into low-malignant and high-malignant forms based on histologic criteria. The malignancy grade assigned to all cytological specimens based on Camus’ classification system was compared with the histopathologically assigned grade of excised tumors based on Kiupel’s classification system. The use of a cytological classification system of MCTs, dividing them into low and high grade, has been investigated in a few studies [7,8,16], which report a sensitivity of 88 to 92% and a specificity of 85% to 94%. Camus’ classification system’s overestimation of high-grade cases, which could result in a more severe course of therapy, is one of its weaknesses [18]. An attempt to overcome the uncertainty of cytological grading has been published by Paes et al.; the authors proposed the simultaneous assessment of the tumor microenvironment and advocated for the incorporation of fibroblasts and/or collagen fibrils in cytologic grading schemes [19]. In well-differentiated MCTs, our observations on H1 histological samples indicated that the connective tissue primarily comprised the pre-existing stroma layer. On the contrary, MCTs with low differentiation exhibited a delicate surrounding connective tissue.

Hergt et al. proposed criteria for a cytological grading system for MCTs that divides tumors into low and high grade. The authors concluded that while this classification is beneficial for the initial assessment, the current consensus suggests that the reliability of cytology is considered insufficient [8]. Lee et al. [20] evaluated the predictive rate of FNA cytology from a previous surgical site for recurrence in incompletely excised MCTs and concluded that the negative predictive value of this technique was 93.5% with an overall accuracy of 88.9%. Based on our findings, the prognostic efficacy for discerning highly malignant MCTs from cytological samples was found to be relatively low, with only 78.57% (out of 14 highly malignant MCTs, correct cytologic grading was achieved in 11) of the MCTs confirmed to be highly malignant through histopathological examination identified as highly malignant through cytology. The degree of malignancy ascribed to cytology samples was verified via histopathology in 33 out of the 45 MCT cases. Among the 12 misclassified cases, cytology overestimated the degree of malignancy in 9 cases and underestimated it in the remaining 3 cases.

When examining the correlation between the Kiupel’s grading system and the cytologic grading system, it was observed that out of the 45 cases analyzed, 3 (accounting for 6.6%) were identified as cytologically low grade but histopathologically high grade (false negatives). Additionally, 9 cases (20%) were found to be cytologically high grade but histopathologically low grade (false positives). False negatives were observed in the studies conducted by Hergt et al. [8] and Scarpa et al. [16], in which 2 out of 38 (5%) and 5 out of 105 (4.8%) cytologically low-grade MCTs displayed histopathological high-grade characteristics, respectively. Correlations between cytological findings and histopathological classifications have also been reported in various other types of neoplasms. Notably, Khan et al. identified six distinct cytological findings that demonstrated a strong correlation with histopathological grade in in situ breast duct carcinomas [21].

Even though histopathological examination requires invasive sampling, it is an indispensable component that cannot be overlooked or disregarded. The grading based on histopathological findings remains an essential cornerstone and is widely regarded as the most accurate approach to predicting the progression of the disease. However, considering the intricacies involved in managing canine MCTs, the incorporation of specific markers is imperative. The histopathologic grading of MCTs in canines is predominantly utilized in conjunction with proliferative markers. While several studies have explored the utilization of immunohistochemistry to investigate markers that aid in the differential diagnosis and enhance our understanding of the tumor’s biological behavior, only a handful of antibodies have demonstrated predictive or prognostic significance. The most widely used immunohistochemical markers in the prognostic evaluation of MCT are Ki-67, AgNOR staining, and KIT pattern [10,22,23,24]. Specifically for MCTs, distinct cut-off values have been proposed for both markers (Ki-67 and Ki-67 × AgNOR) on histological slides. For example, for the Ki-67 marker, a mean score greater than or equal to 23 (immunopositive nuclei/100 cells) has been used for the classification of the neoplasm as highly malignant. Similarly, the cut-off value of 54 for the product Ki-67 × AgNOR has been used to classify the tumor as having high or low malignancy [6,10].

According to Kiupel and Camus (2019), a significant challenge in evaluating cell proliferation markers in canine cutaneous MCTs arises from the absence of standardized evaluation methods and, more crucially, the lack of a clear definition of the area to be assessed [13]. Regions exhibiting more pronounced cellular activity or signaling are deemed more suitable for evaluation. These challenges become even more pronounced when assessing cytological preparations, which typically contain a smaller cell population compared to histopathological samples. In this study, the estimation of Ki-67 involved calculating the number of immunopositive cells out of a total of 100 cells, a metric commonly employed for calculating the Ki-67 index in histological samples [25].

The AgNOR staining technique elucidated in this study, parallel to those of a recent study conducted by Mann [26], achieved satisfactory results in cytological slides, thus permitting a swift and effortless determination of the degree of malignancy in MCTs. The value of AgNOR staining in both cytological and histopathological specimens has been examined by Vajdovich et al. (2004). Their research pertained to cases of canine lymphoma, demonstrating that measurements of AgNORs in both cytological and histological samples exhibited statistically significant disparities between control dogs and dogs afflicted with lymphoma [27]. Similarly, Kravis et al. found an analogous linear correlation in the quantification of AgNORs between cytological and histopathological specimens in dogs with MCTs. Furthermore, their findings demonstrate a correlation between the measurement of AgNORs in cytological specimens and the Patnaik histological grade of the neoplasm [28]. Immunocytochemistry represents an advanced diagnostic technique utilized within the realm of veterinary cytology, albeit not as widely employed as immunohistochemical and histochemical methods [29]. In the veterinary literature, the evidence of the utilization of proliferative indicators on cytological specimens for the diagnosis of canine MCTs is exceedingly limited, with no available data assessing the implementation of immunocytochemical and cytochemical methods for the cytological classification of MCTs. In a comprehensive study, the distribution of CD117 immunocytochemistry staining patterns in canine MCT smears was examined [30]. The authors revealed that perimembrane staining (KIT pattern I) was solely expressed in low-grade tumors, whereas perinuclear (KIT pattern II) and diffuse cytoplasmic (KIT pattern III) staining were exclusively observed in high-grade MCTs.

To the authors’ knowledge, the present study represents the first known examination of the immunocytochemical prognostic capacity for canine cutaneous MCTs. Up until now, no previous research has provided data regarding the establishment of cut-off values for Ki-67 and Ki-67 × AgNOR markers in cytological samples. These values, which have hitherto only been determined for histological samples, were determined within the framework of this study, and samples were consequently classified as either demonstrating low or high malignancy.

The utilization of both markers in this study marginally reduced the occurrence of inaccurate positive cases, while it did not alter the number of inaccurate negatives, an observation that likely enhances their secure application in MCT smears alongside cytopathological grading. Inaccurate negative cases may potentially complicate surgical treatment more than inaccurate positives as the latter tends to prompt more cautious therapeutic strategies [4,5,6]. The three false negative cases misclassified as low-grade MCTs by cytology (Table 1 and Table S5) were classified correctly as high grade when we applied Ki-67 and Ki-67 × AgNOR or Ki-67 × AgNOR alone. Ki-67 and Ki-67 × AgNOR classifications correctly predicted high grade in 2/3 of those cases. In the third case, only Ki-67 × AgNOR classified it correctly as high; however, Ki-67 value was near the cut-off value of 6.5 (6.00).

The proposed cut-off values for the Ki-67 and Ki-67 × AgNOR proliferation markers, as indicated in this study, aim to enhance the predictive prognostic capability of cytology when analyzing samples from cutaneous mast cell tumors (MCTs). Although the rate of predicting histopathological grade did not change significantly, for the few cases where the malignancy level was accurately identified with the aid of immunocytochemistry, a deeper understanding of the tumor’s biological behavior was gained. This revelation holds even greater importance when considering individualized care of canine patients rather than solely evaluating the efficacy of diagnostic systems. Inaccurate grading of a single mast cell tumor (MCT) case has the potential to result in life-threatening complications for the patient [15,22,31].

A limitation of the study is the inadequate representation of highly malignant mast cell tumors (MCTs) as only 14 out of the 45 cases were classified as such. Given that highly malignant MCTs are statistically less prevalent—with around 90% of cutaneous MCTs being low grade, as indicated by the study that established the two-tier system [5,9]—we considered this distribution to be reflective of a common occurrence in the broader population. It is possible that within a wider range of MCTs, there could be a different determination of Ki-67 and Ki-67 × AgNOR cut-off values.

Finally, for immunocytochemical and cytochemical methods to be used routinely in the diagnosis of cutaneous MCTs in dogs, the cut-off values for cell proliferation markers should be confirmed in additional studies and directly correlated with survival rates, recurrence probability, and metastasis occurrence.

## 5. Conclusions

All considered, cytological grading of canine MCTs is a helpful prognostic tool, particularly in conjunction with the proliferative markers Ki-67 and Ki-67 × AgNOR in smears. However, prognostic studies on larger caseloads assessing the reproducibility of this combined grading system and direct correlation with survival rates would be beneficial to enhance its function in a diagnostic routine.

## Figures and Tables

**Table 1:** Number of low- and high-grade MCTs classified according to Kiupel’s system, Camus’s system, Ki-67, and Ki-67 × AgNOR as well as the misclassified cases.

Grading Systems Markers	Grade	Cases	Misclassified Cases
Kiupel’s histopathological system	Low	31/45 (68.9%)	-
	High	14/45 (31.1%)	-
Camus’s cytological system	Low	25/45 (55.6%)	3/25
	High	20/45 (44.4%)	9/20
Ki-67 grading in the present study	Low	26/45 (57.8%)	3/26
	High	19/45 (42.2%)	8/19
Ki-67 × AgNOR grading in the present study	Low	27/45 (60%)	3/27
	High	18/45 (40%)	7/18

## Data Availability

All data generated or analyzed during this study are included in this published article. The datasets used and/or analyzed during the present study are available from the first author upon reasonable request.

## Supplementary Materials

The following supporting information can be downloaded at: https://www.mdpi.com/article/10.3390/vetsci11010023/s1, Table S1: Cytological grading allocated in C1 samples and histopathological grading of samples H2. Table S2: Ki-67, AgNORs, and Ki-67 × AgNOR values in the C1 samples of the MCTs included in the study. Table S3: Cut-off values, sensitivity, and specificity for the Ki-67 index in C1 samples of the MCTs included in the study. Table S4: Cut-off values, sensitivity, and specificity for the Ki-67 × AgNOR index in C1 samples of the MCTs included in the study. Table S5: Cytological, histopathological, Ki-67, and Ki-67 × AgNOR gradings of all 45 MCT cases.
