# Supplementary material for: Cutaneous Canine Mast Cell Tumor: The Use of Proliferative Markers (Ki-67 and Ki-67 × AgNOR) in Cytological Samples for Diagnosis and Prognosis
Source: Vet Sci. 2024 Jan 7;11(1):23. doi: 10.3390/vetsci11010023 (PMC10821150; doi:10.3390/vetsci11010023)
Supplement: Supplementary file 1 [file vetsci-11-00023-s001.zip › vetsci-2795559-supplementary.pdf]

SUPPLEMENTARY MATERIALS

Table S1. Cytological grading allocated in samples C1 and histopathological grading of samples H2.

	**Cytological Grading**	**Histopathological Grading**
**No**	**C1**	**H2**
**1***	High	Low
2	Low	Low
3	High	High
4	High	High
5	High	High
**6**	Low	High
7	Low	Low
8	High	High
9	Low	Low
10	High	High
11	Low	Low
**12**	High	Low
13	Low	Low
14	Low	Low
15	Low	Low
16	Low	Low
**17**	High	Low
**18**	High	Low
19	Low	Low
**20**	Low	High
21	Low	Low
22	High	High
**23**	Low	High
24	High	High
25	High	High
26	Low	Low
27	Low	Low
28	High	High
**29**	High	Low
**30**	High	Low
31	Low	Low
32	High	High
33	High	High
**34**	High	Low
35	Low	Low
36	Low	Low
37	Low	Low
38	Low	Low
39	Low	Low
**40**	High	Low

41	Low	Low	5
42	Low	Low	
43	Low	Low	6
44	Low	Low	
**45**	High	Low	7
			8

**Bold: cases with grade discrepancy*

Table S2. Ki-67, AgNORs and Ki-67 × AgNOR values in the C1 samples of the MCTs included in the study.

	Average values		
**No**	**Ki-67**	***AgNORs***	**Ki67 × AgNOR**
1	3	*1.74*	5.22
2	2	*1.45*	2.90
3	7	*1.53*	10.71
4	9	*2.71*	24.39
5	2	*2.05*	4.10
6	8	*2.39*	19.12
7	1	*2.37*	2.37
8	16	*4.53*	72.48
9	7	*1.82*	12.74
10	29	*3.21*	93.09
11	9	*1.87*	16.83
12	8	*2.05*	16.40
13	3	*1.79*	5.37
14	9	*2.58*	23.22
15	7	*2.03*	14.21
16	3	*1.79*	5.37
17	7	*2.18*	15.26
18	1	*1.32*	1.32
19	5	*3.39*	16.95
20	6	*2.61*	15.66
21	1	*1.82*	1.82
22	11	*2.68*	29.48
23	7	*2.89*	20.23
24	13	*1.61*	20.93
25	12	*3.08*	36.96
26	2	*2.13*	4.26
27	9	*2.26*	20.34
28	4	*3.47*	13.88
29	6	*3.03*	18.18
30	1	*2.24*	2.24
31	3	*3.13*	9.39
32	8	*2.79*	22.32
33	17	*1.87*	31.79

34	4	*2.34*	9.36
35	2	*2.74*	5.48
36	5	*1.89*	9.45
37	9	*1.92*	17.28
38	4	*2.50*	10.00
39	1	*1.34*	1.34
40	4	*2.24*	8.96
41	6	*2.21*	13.26
42	3	*2.05*	6.15
43	2	*2.61*	5.22
44	1	*3.11*	3.11
45	4	*2.79*	11.16

Table S3. Cut-off values, sensitivity and specificity for the Ki-67 index in C1 samples of the MCTs included in the study.

Test Result Variable(s)	Cut off points	Sensitivity	1-Specificity
Ki-67	.0000	1.000	1.000
	1.5000	1.000	.806
	2.5000	.929	.677
	3.5000	.929	.516
	4.5000	.857	.387
	5.5000	.857	.323
	**6.5000***	**.786**	**.258**
	7.5000	.643	.161
	8.5000	.500	.129
	10.0000	.429	.000
	11.5000	.357	.000
	12.5000	.286	.000
	14.5000	.214	.000
	16.5000	.143	.000
	23.0000	.071	.000
	30.0000	.000	.000

**Cut-off value chosen for Ki-67 and the sensitivity and specificity value it presented are marked in bold.*

Table S4. Cut-off values, sensitivity and specificity for the Ki-67 × AgNOR index in C1 samples of the MCTs included in the study.

Test Result Variable(s)	Cut off points	Sensitivity	1-Specificity
Ki-67 × AgNOR	.3200	1.000	1.000
	1.3300	1.000	.968
	1.5800	1.000	.935
	2.0300	1.000	.903

	2.3050	1.000	.871
	2.6350	1.000	.839
	3.0050	1.000	.806
	3.6050	1.000	.774
	4.1800	.929	.774
	4.7400	.929	.742
	5.2950	.929	.677
	5.4250	.929	.613
	5.8150	.929	.581
	7.5550	.929	.548
	9.1600	.929	.516
	9.3750	.929	.484
	9.4200	.929	.452
	9.7250	.929	.419
	10.3550	.929	.387
	10.9350	.857	.387
	11.9500	.857	.355
	13.0000	.857	.323
	13.5700	.857	.290
	14.0450	.786	.290
	14.7350	.786	.258
	**15.4600***	**.786**	**.226**
	16.0300	.714	.226
	16.6150	.714	.194
	16.8900	.714	.161
	17.1150	.714	.129
	17.7300	.714	.097
	18.6500	.714	.065
	19.6750	.643	.065
	20.2850	.571	.065
	20.6350	.571	.065
	21.6250	.500	.032
	22.7700	.429	.032
	23.8050	.429	.000
	26.9350	.357	.000
	30.6350	.286	.000
	34.3750	.214	.000
	54.7200	.143	.000
	82.7850	.071	.000
	940900	.000	.000

**Cut-off value chosen for Ki-67 × AgNOR and the sensitivity and specificity value it presented are marked in bold.*

Table S5 Cytological, histopathological, Ki-67 and Ki-67 × AgNOR gradings of all 45 MCT cases.

**Cases**	CYTOLOGIC GRADING CAMUS	**HISTOPATHOLOGIC GRADING KIUPEL**	Ki-67 count	Ki-67 GRADING **6,5**	Ki-67xAGNOR scorings	Ki-67 x AGNOR GRADING **15,46**
**1**	High	**Low**	3,00	Low	5,22	Low
**2**	Low	**Low**	2,00	Low	2,90	Low
**3**	High	**High**	7,00	High	10,71	Low
**4**	High	**High**	9,00	High	24,39	High
**5**	High	**High**	2,00	Low	4,10	Low
**6**	Low	**High**	8,00	High	19,12	High
**7**	Low	**Low**	1,00	Low	2,37	Low
**8**	High	**High**	16,00	High	72,48	High
**9**	Low	**Low**	7,00	High	12,74	Low
**10**	High	**High**	29,00	High	93,09	High
**11**	Low	**Low**	9,00	High	16,83	High
**12**	High	**Low**	8,00	High	16,40	High
**13**	Low	**Low**	3,00	Low	5,37	Low
**14**	Low	**Low**	9,00	High	23,22	High
**15**	Low	**Low**	7,00	High	14,21	Low
**16**	**Low**	**Low**	**3,00**	**Low**	**5,37**	Low
**17**	High	**Low**	7,00	High	15,26	Low
**18**	High	**Low**	1,00	low	1,32	Low
**19**	Low	**Low**	5,00	Low	16,95	High
**20**	Low	**High**	6,00	Low	15,66	High
**21**	Low	**Low**	1,00	Low	1,82	Low
**22**	**High**	**High**	**11,00**	**High**	**29,48**	High
**23**	Low	**High**	7,00	High	20,23	High
**24**	High	**High**	13,00	High	20,93	High
**25**	High	**High**	12,00	High	36,96	High
**26**	**Low**	**Low**	**2,00**	**Low**	**4,26**	Low
**27**	Low	**Low**	9,00	High	20,34	High
**28**	High	**High**	4,00	Low	13,88	Low
**29**	High	**Low**	6,00	Low	18,18	High
**30**	High	**Low**	1,00	Low	2,24	Low
**31**	Low	**Low**	3,00	Low	9,39	Low
**32**	High	**High**	8,00	High	22,32	High
**33**	High	**High**	17,00	High	31,79	High
**34**	High	**Low**	4,00	LOW	9,36	Low
**35**	Low	**Low**	2,00	LOW	5,48	Low
**36**	Low	**Low**	5,00	Low	9,45	Low
**37**	Low	**Low**	9,00	High	17,28	High
**38**	Low	**Low**	4,00	Low	10,00	Low

**39**	Low	**Low**	1,00	Low	1,34	Low
**40**	High	**Low**	4,00	Low	8,96	Low
**41**	Low	**Low**	6,00	Low	13,26	Low
**42**	Low	**Low**	3,00	Low	6,15	Low
**43**	Low	**Low**	2,00	Low	5,22	Low
**44**	Low	**Low**	1,00	Low	3,11	Low
**45**	High	**Low**	4,00	Low	11,16	Low
